# Exploring the Rare Occurrence of Giant Cell Tumour in Distal Tibia

**DOI:** 10.5704/MOJ.2511.013

**Published:** 2025-11

**Authors:** k Parakh-Naman, N Chauhan, R Mishra

**Affiliations:** 1 Department of Orthopaedics, Teerthanker Mahaveer Medical College and Research Centre, Moradabad, India; 2 Department of Orthopaedics, Rama Medical College, Hospital and Research Centre, Hapur, India; 3 Department of Orthopaedics, Maulana Azad Medical College (MAMC), New Delhi, India

**Keywords:** giant cell tumour, aggressive lesion, distal tibia, tumour resection, ankle arthrodesis

## Abstract

**Introduction:**

Giant cell tumours (GCT) of the distal tibia are uncommon, locally aggressive lesions that are frequently difficult to manage due to the anatomical and functional intricacies of the region. This case study seeks to elucidate the clinical presentation, surgical procedures, and results in three individuals with distal tibial giant cell tumours, highlighting management and reconstructive techniques in this uncommon location with higher risk of morbidity compared to other locations and to achieve pain free ambulation.

**Materials and methods:**

Three cases presenting with localised pain and swelling with painful weight-bearing in the distal tibia, were included in the study. Two patients had primary GCT whereas one had recurrence of the tumour after being managed elsewhere. All patients had biopsy and radiologically proven GCT with differing degrees of local aggressiveness. The management strategy involved wide-margin tumour resection followed by reconstruction techniques including contralateral fibular strut grafting and ankle arthrodesis with cementation and bone graft to restore structural integrity and functionality.

**Results:**

All three patients underwent successful tumour resection and reconstruction without major intra-operative complications. Radiological assessments confirmed adequate union of grafts and no signs of recurrence during follow-up.

**Conclusion:**

This series highlights the efficacy of various modalities for the treatment of GCT of distal tibia. While these methods demonstrate promising results in terms of stability and patient satisfaction, vigilance for recurrence and long-term complications remains critical.

## Introduction

Giant cell tumours (GCT) account for nearly 5% of all tumours of the bone and 22% of all benign bone tumours. It affects the meta-epiphyseal regions of the bone, most commonly affecting the distal femur, proximal tibia and distal radius^1^. It is a locally aggressive tumour with a female preponderance and majority of these seen in the third decade of life. Secondary forms may be seen in severe, chronic Paget’s disease of the bone (PDB) and these present after the fourth decade of life^2^. Rarely, they may present as a malignant tumour and can cause pulmonary metastasis3.

Radiographically, it presents as a well-defined, purely osteolytic lesion with cortical thinning and endosteal scalloping. On MRI, GCT shows a low/intermediate signal on T1 and high signal on T2 weighted images. Differential diagnoses include chondroblastoma, Aneurysmal Bone Cyst (ABC), Simple Bone Cyst (SBC), giant cell dominant Osteosarcoma and brown tumour of hyperparathyroidism^3,4^. Campanacci classified GCT radiologically into three grades - Grade 1 (Latent) was a well-defined lesion with no cortical involvement, Grade 2 (Active) consisted of a well-defined lesion with cortical thinning and in Grade 3 (Aggressive), the lesion had indistinct edges with soft tissue infiltration and cortical erosion^5^.

Surgery remains the mainstay of treatment in GCT. Surgical options include intralesional resection using curettage in combination with local adjuvants like bone grafts, cementation, liquid nitrogen or impregnation with phenol, zinc chloride or hydrogen peroxide. Radiotherapy is useful when surgery is contraindicated. Denosumab is a human monoclonal antibody that targets RANKL, thereby inhibiting osteoclast activity and bone resorption. This is particularly helpful pre-operatively in large aggressive tumours to reduce their size, solidify the reactive rim and make resection easier.

It can also convert a Grade 3 lesion into a more manageable Grade 2 lesion, allowing for curettage instead of wide resection. Bisphosphonates although less effective than denosumab, can also be used as an anti-resorptive agent^2^. The location presents its own unique challenges and consideration due to its proximity to the ankle joint. It warrants a vigilant approach to prevent significant morbidity as compared to other sites and ensure pain free ambulation post-operatively.

This study shows treatment of GCT of distal tibia in three patients managed by three different surgical manoeuvres. We aim to acknowledge the challenges in choosing the correct operative procedure in cases with various different clinico-radiological presentations.

## Materials and Methods

A total three patients presenting with GCT of the distal tibia were included in this case series (Table I). Ethical clearance was obtained from the Institutional Ethical Committee (IEC) along with a written informed consent from all patients in the study.

**Table I:** Characteristics of the Patient Population and summary of procedure done (AP: Antero-Posterior, TR: Transverse, CC: Craniocaudal).

S. No.	Age	Side	Extent	Campanacci Grade	Tumour Size (Ap*Tr*Cc) In Mm	Duration (In Months)	Surgical Procedure	Complication
1	22	Left	Subarticular	2	35*30*22	12	Tumour Curettage + Adjuvant Therapy + Autologous Bone Graft + Bone Cement	None
2	37	Right	Articular	3	52*53*61	18	En Bloc Resection + Adjuvant Therapy + Ankle Arthrodesis Using Contralateral Fibular Strut Graft and Distal Tibia LCP + Bone Grafting	None
3	38	Left	Articular	2	57*58*41	6	Extended Curettage + Adjuvant Therapy + Ankle Arthrodesis with TTC Nail + Bone Grafting	none

**Fig. 1:**
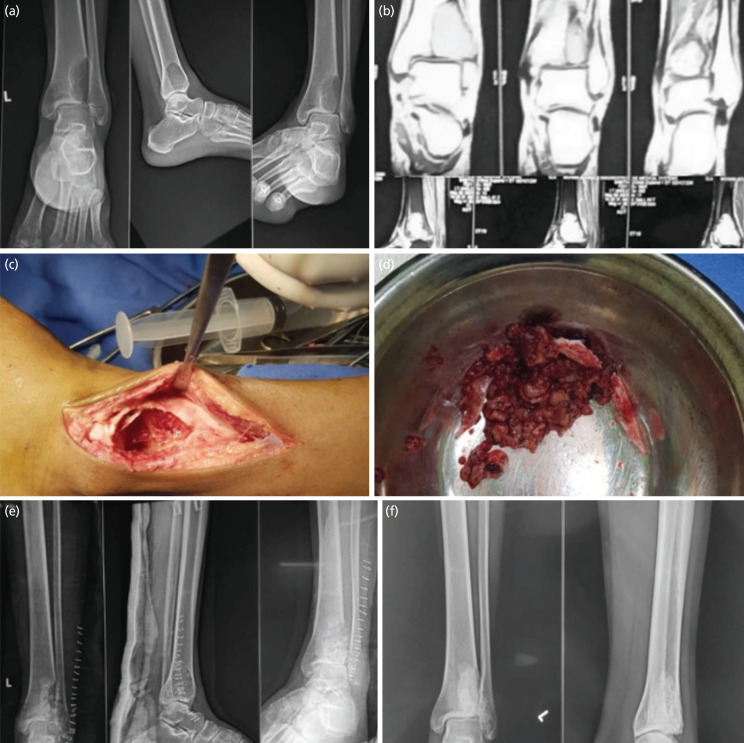
(a) Radiograph of the involved ankle showing a lytic lesion at the tibiofibular syndesmotic interface extending into the subarticular region of the distal tibia. (b) Magnetic Resonance Imaging of the involved ankle confirming the diagnosis of GCT. (c) Intra-operative image after proper curettage and chemical treatment of the tumour. (d) Intra-operative picture of the curetted tumour tissue (e)post operative radiograph after cementation of the defect. (f) One year post-operative radiograph.

Patient 1 was a 22-year-old female presented to the Outpatient Department (OPD) with complains of pain and swelling over left ankle and foot for one year. She also had complained of pain on walking long distances. The patient took treatment from multiple quacks with only symptomatic relief in pain. Clinical examination, radiographs and MRI confirmed the presence of GCT in the distal tibia left side. The patient was managed surgically by curettage and evacuation of the tumour tissue along with local instillation of hydrogen peroxide. The bony defect was filled with bone cement. Patient was advised below knee slab and assisted Non-weight Bearing (NWB) walk for six weeks and partial to full weight bearing thereafter. The patient was followed-up regularly and showed significant improvement in pain and ankle motion. Twelve-month follow-up radiograph showed satisfactory cement uptake with no evidence of tumour recurrence (Fig. 1). Patient was able to squat and had painless ankle range of motion.

**Fig. 2:**
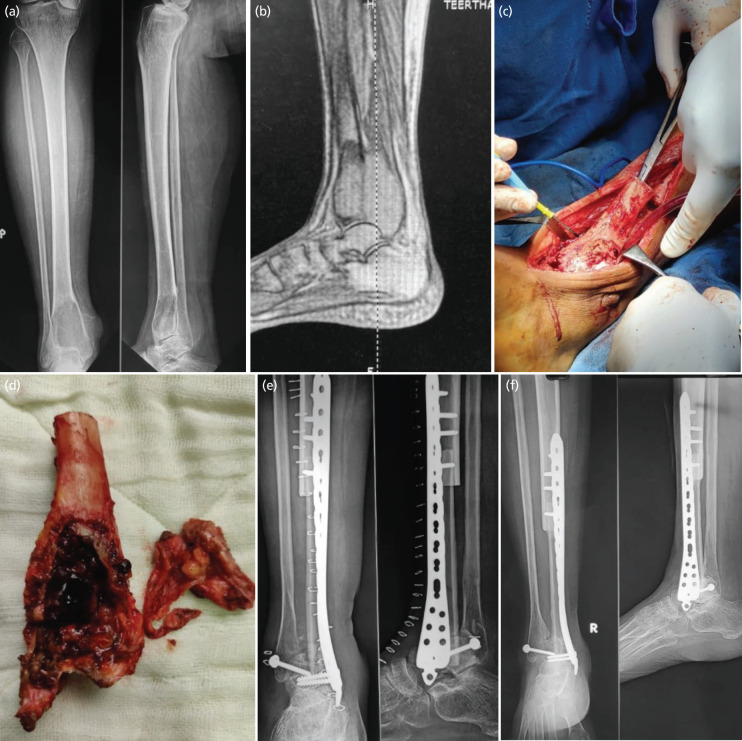
(a) Radiograph of the patient on presentation showing an expansile lytic lesion in the distal tibia involving the articular surface. (b) Magnetic Resonance Imaging confirming GCT and its soft tissue extension. (c) Intra-operative image of en bloc wide resection of the distal tibia tumour. (d) Resected distal tibia with tumour tissue. (e) Post-operative radiograph showing arthrodesis of the ankle using autologous fibular strut graft and fixation using a medial distal tibial Locking Compression Plate. (f) Two year postoperative radiograph showing radiological union at the proximal and distal graft sites.

Patient 2 was a 37-year-old female presented to the OPD with complains or pain and swelling in her Right ankle with difficulty in bearing weight over right lower limb since 18 months. The patient had history of treatment taken from multiple local doctors and quacks but with only brief symptomatic relief. Radiographs and MRI were performed which confirmed the diagnosis of Campanacci Grade 3 GCT. The patient was treated with en-bloc resection of the tumor affected distal tibia along with fixation using the contralateral fibular strut graft and Medial distal tibial Locking Compression Plate (LCP) and bone grafting to achieve arthrodesis of the affected ankle. The resected bone was sent for histopathological examination which further confirmed the diagnosis of GCT. Patient was discharged with advice for assisted NWB walk for six weeks and thereafter advised weight bearing as tolerated. Follow-up radiographs were done on each visit. Eighteen months post-operative radiograph showed union and proximal and distal graft sites with good functional outcome and no complications (Fig. 2).

**Fig. 3:**
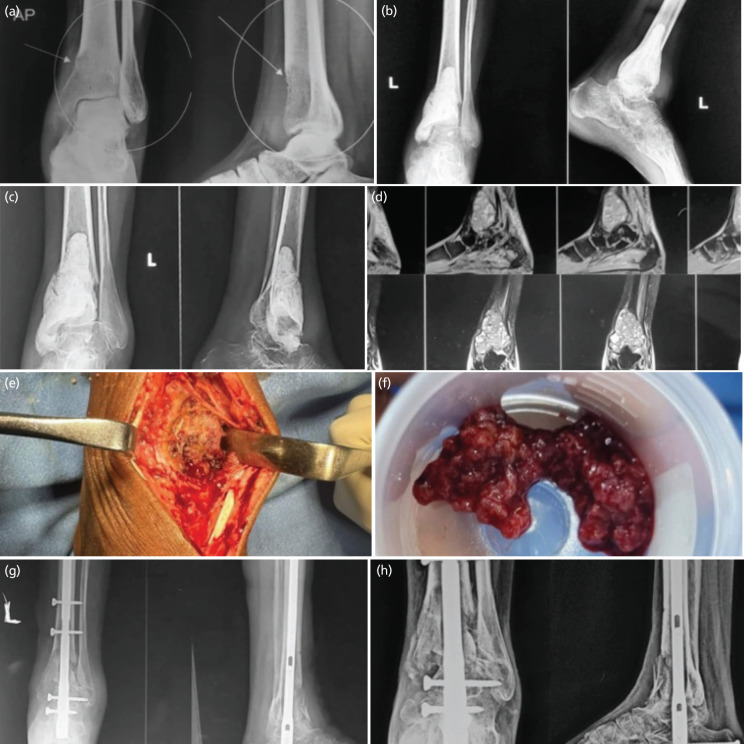
(a) Radiograph showing a lytic lesion in the distal tibia with subarticular extension. (b) Post-operative radiograph after curettage and bone cementation. (c) One year post-operative radiograph with signs of tumour recurrence. (d) Magnetic Resonance Imaging confirming the recurrence of tumour with soft tissue extension. (e) Intra-operative image of the tumour. (f) Intra-operative image of the curetted tumour. (g) Post-operative radiograph showing ankle arthrodesis using a retrograde locked intramedullary nail with autologous iliac crest bone graft. (h) Six months post-operative radiograph.

Patient 3 was a 38-year-old female presented to us with complains of pain in the left ankle since six months. The patient was managed surgically elsewhere for GCT of the distal tibia six months back. Radiographs available indicated that treatment was done by curettage and cementation of the defect. Clinical examination, radiographs and MRI confirmed the recurrence of tumour. The patient was managed by extended curettage after removal of the cement and local treatment with hydrogen peroxide followed by retrograde intramedullary nailing of the ankle using a Tibio TaloCalcaneum (TTC) Nail along with local bone grafting from ipsilateral iliac crest cortico-cancellous bone graft to achieve fusion (arthrodesis) of the ankle and subtalar joints. Post-operatively, below knee slab was given along with advice for assisted NWB walk for six weeks and partial to full weight bearing as pain allowed thereafter. The patient was followed-up regularly and showed significant improvement in pain. Six months post-operative radiograph showed signs of graft uptake with no complications (Fig. 3).

## Results

The diagnosis in all three patients was further confirmed using Histopathological examination by staining which confirmed the presence of multinucleated giant cells and mononuclear stromal cells with an absence of osteoid or cartilage formation. Variable mitotic activity in the stromal cells along with hemosiderin deposition further confirmed the diagnosis of GCT in our patients. The diagnosis could have been further strengthened by using Immunohistochemistry which was not possible at our centre.

## Discussion

GCT of the distal tibia has a rare occurrence (2 – 4% of all primary GCTs). The tumour location in proximity to the ankle and its critical weight bearing function necessitates careful planning for surgical intervention. This site predisposes patients to significant morbidity due to severe pain during ambulation and even at rest^6^.

Campanacci Grade 1 and 2 tumours are best managed by aggressive curettage along with adjuvants like phenol or hydrogen peroxide. The defect can later be filled with Polymethylmethacrylate (PMMA) or hydroxyapatite. Grade 3 tumours are treated with en bloc resection of the tumour^7^.

Recently, a number of treatment options for the management of bone defect after tumour resection in GCT of distal tibia have been postulated. The biological methods include the use of autograft (fibular strut), allograft, tumour recycled bone or distraction osteogenesis whereas the non-biological methods include prosthetic replacement using standard or custom-made 3D printed implants. Amputation remains the last resort in treatment of recurrent cases with poor functional status^8^.

Prosthetic replacement requires ample muscle coverage and adequate bone stock while carrying the risk of deep infection, implant loosening and talar collapse. Distraction osteogenesis is prone to non-union at the docking site and pin or wire-tract infection. Hence in tumours with subarticular or articular involvement, ankle arthrodesis provides good ankle stability and bone union along with better post-operative function^8^.

Tumour recurrence remains a major concern among other complications in management of GCT. While overall recurrence rates for GCT range from 20% to 50% depending upon the treatment modality and tumour stage, specific data for distal tibia GCT are less extensively reported. Su *et al* conducted a study on 87 cases of GCT in 2004 which were managed either by curettage and adjuvants or wide excision. The recurrence rate in their study was 12.6%, mostly seen in the cases managed with curettage. Thirteen patients showed other complications^9^.

Although none of our patients showed any complications or evidence of recurrence, those in other studies could be attributed to the lack of good muscle coverage, unique biomechanical stresses on the distal tibia, potential for inadequate removal due to anatomical constraints and proximity to neurovascular structures.

The management plan in this case series was affected by the articular extent of the tumour. Both patients with articular surface involvement were managed with arthrodesis of the ankle joint either with a plate and fibular strut graft or with a TTC Nail and iliac crest bone graft. The patient with subarticular tumour was managed by curettage and treatment with hydrogen peroxide and phenol and bone cementation of the defect.

A vigilant approach is required in diagnosis and treatment of the tumour and evaluating for pulmonary metastasis pre-operatively. Unlike GCTs in more proximal locations, extensive resection in distal tibia can significantly compromise ankle function and stability, potentially leading to long term disability. Hence, a cautious approach is needed in these cases, balancing tumour eradication with functional preservation.

Denosumab can be used in the pre- and post-operative phase to reduce the size and progression of the tumour and ensure re-ossification and mineralisation of the affected bone and increasing the intralesional bone density in case of unresectable or recurrent tumours^4^.

Radiation therapy is reserved and kept as a last resort in distal tibia GCT where surgical resection is incomplete, carries unacceptable morbidity or for recurrent cases that are not amenable for further surgery. Its use is limited by the risk of malignant transformation (secondary osteosarcoma) in the irradiated field, especially with higher doses^10^.

Further multicentre studies with larger cohorts are needed to establish definitive guidelines for optimal treatment strategies, evaluate long term functional outcomes, and explore the role of novel therapeutic agents in the treatment of GCT in this challenging anatomical site.

The drawbacks of this study would include a small group of patients and a relatively short duration of follow-up. The study could have also been strengthened by the use of a scoring system to assess the functional outcomes.

## Conclusion

This series highlights the efficacy of various modalities for the treatment of GCT of the distal tibia. The efficacy of the various modalities demonstrated promising results in terms of stability and patient satisfaction. However, the vigilance for recurrence and long-term complications remains critical.

## References

[1] Agrawal AC, Ojha MM, Banerjee S. (2023). Tumor Resection, Reconstruction, and Ankle Fusion for Recurrent Giant Cell Tumor of the Distal Tibia.. J Orthop Trauma Rehabil.

[2] Scotto di Carlo F,, Whyte MP,, Gianfrancesco F. (2020). The two faces of giant cell tumor of bone.. Cancer Lett..

[3] Kapil Mani KC, Raju GC (2022). Giant cell tumor of distal tibia in an immature skeleton: A case report.. J Orthop Rep..

[4] Parmeggiani A, Miceli M, Errani C, Facchini G (2021). State of the Art and New Concepts in Giant Cell Tumor of Bone: Imaging Features and Tumor Characteristics.. Cancers (Basel).

[5] Campanacci M, Baldini N, Boriani S, Sudanese A (1987). Giant-cell tumor of bone.. J Bone Joint Surg Am.

[6] Bhaskar Reddy KV,, Chary NB,, Mogili CK. (2021). Surgical management of giant cell tumor in adolescent by excision or curettage followed by fibular strut graft.. Int J Res Orthop..

[7] Tsukamoto S, Mavrogenis AF, Kido A, Errani C (2021). Current Concepts in the Treatment of Giant Cell Tumors of Bone.. Cancers (Basel).

[8] Zhao Z, Yan T, Guo W, Yang R, Tang X, Wang W (2018). Surgical options and reconstruction strategies for primary bone tumors of distal tibia: A systematic review of complications and functional outcome.. J Bone Oncol.

[9] Napoli R, Mukherjee A, Rossi M (2024). Distal tibia giant cell tumor surgical treatment: A case report.. FASTRAC.

[10] Su YP, Chen WM, Chen TH (2004). Giant-cell tumors of bone: an analysis of 87 cases.. Int Orthop.

